# Management and outcomes of chest-indrawing pneumonia among children aged 2–59 months in a programme setting in Ethiopia: a prospective observational study

**DOI:** 10.7189/jogh.15.04217

**Published:** 2025-08-04

**Authors:** Zemene Tigabu, Alemayehu Teklu Toni, Tadesse Guadu, Tesfahun Melese Yilma, Tadesse Awoke, Garedew Tadege Engdaw, Ashenafi Tazebew, Shamim Ahmad Qazi, Yasir Bin Nisar

**Affiliations:** 1 Department of Pediatrics and Child Health, School of Medicine, College of Medicine and Health Science, University of Gondar, Gondar, Ethiopia; 2 Department of Environmental and Occupational Health, and Safety, Institute of Public Health, University of Gondar, Gondar, Ethiopia; 3 Department of Health Informatics, College of Medicine and Health Sciences, University of Gondar, Gondar, Ethiopia; 4 Department of Epidemiology and Biostatistics, Institute of Public Health, College of Medicine and Health Sciences, University of Gondar, Gondar, Ethiopia; 5 Independent consultant, Geneva, Switzerland; 6 Department of Maternal, Newborn, Child and Adolescent Health and Ageing, World Health Organization, Geneva, Switzerland

## Abstract

**Background:**

Pneumonia is a leading cause of morbidity and mortality in children under five years of age. In 2012, the World Health Organization revised its guidelines for managing childhood pneumonia and recommended oral amoxicillin for the outpatient treatment of chest indrawing pneumonia in children aged 2–59 months. While the Ethiopian government subsequently adopted these revised pneumonia guidelines, the level of their implementation and of the related treatment outcomes remains less known. We aimed to determine the outcomes of this approach at primary healthcare facilities in Ethiopia.

**Methods:**

We conducted a prospective, observational cohort study at five health centres in Northern Ethiopia from November 2022 to November 2023. Trained health workers screened all children aged 2–59 months who had cough or difficult breathing and managed them according to the integrated management of childhood illness chart booklet. Children with chest indrawing pneumonia who lived in the study catchment area and whose parents or guardians consented were enrolled. An independent data collector conducted a follow-up visit on day 15 to collect information on their survival status and the treatment received after enrolment. The primary outcome was case fatality risk (CFR), calculated as the proportion of children who died by day 15 after enrolment among all enrolled children.

**Results:**

We screened 3492 children aged 2–59 months, enrolling 345 with chest indrawing pneumonia. All were prescribed oral amoxicillin. The majority (n = 340, 98.6%), received a five-day prescription, while the remaining five were prescribed a seven-day course. We assessed 333 children on day 15 for study outcomes. Twelve (3.5%) were lost to follow-up. Two children died, resulting in a CFR of 0.6 (95% confidence interval = 0.35, 0.85). Most children (n = 315, 94.6%), adhered to the five-day course of amoxicillin, while 18 (5.4%) did not complete the entire course. Thirteen (3.9%) children were taken to a hospital between days two and 15, six received outpatient treatment, and seven were hospitalised. All 13 were alive and well on day 15.

**Conclusions:**

In a programme setting, children aged 2–59 months with chest indrawing pneumonia managed at the primary healthcare facilities on an outpatient basis with oral amoxicillin had low CFR, low hospitalisation rates, and high adherence to treatment.

**Registration:**

ISRCTN: 12687253

Pneumonia remains a leading cause of morbidity and mortality among children under five years of age globally, with the highest burden observed in sub-Saharan Africa and Southeast Asia [1]. The condition caused over 725 000 deaths in this population in 2021 [2] and, according to estimates from the Global Burden of Disease study, accounted for 68 million episodes and 5.13 million hospital admissions in 2016 [3]. Ethiopia ranks fifth among the six high-burden countries with pneumonia-related morbidity and mortality [4], with a high pooled prevalence of 20.7% [5].

To improve the management of pneumonia and other childhood illnesses, the World Health Organization (WHO) and the United Nations Children’s Fund developed the integrated management of childhood illness (IMCI) approach for health workers at primary healthcare facilities [6] and an integrated community case management protocol for community-level health workers [7]. In 2012, the WHO revised the guidelines for managing childhood pneumonia, classifying cases into ‘no pneumonia’ (cough and cold), ‘pneumonia’ (fast breathing or chest indrawing), or ‘severe pneumonia’ (presence of any general danger sign, stridor in a calm child, or oxygen saturation <90%) [8]. The guidelines recommend that children with fast breathing or chest indrawing pneumonia be treated with oral amoxicillin at an outpatient level, while ‘severe pneumonia’ cases are referred to a higher facility for further management [8,9]. These updated recommendations have been implemented through the updated IMCI tool [6] in multiple countries [10], including Ethiopia [11]. The potential benefits of the revised pneumonia guideline include increased access to treatment, expanded treatment coverage, and a reduced financial burden on health systems and families [12,13].

Concerns have arisen regarding the implementation of the revised WHO recommendation, particularly after a study conducted in Kenya reported an increased risk of mortality among children with chest indrawing pneumonia who also presented with pallor and severe wasting [14]. There is also limited empirical evidence on implementing the revised IMCI pneumonia management protocol in primary healthcare facilities, including antibiotic use, treatment adherence, and treatment outcomes such as deterioration, hospital admission, or death, both in Ethiopia and elsewhere.

We aimed to determine the survival status of children aged 2–59 months having chest indrawing pneumonia who presented at primary healthcare facilities in a programme setting and were followed up on day 15. This evidence can facilitate the implementation of pneumonia management recommendations for children in low-resource settings like Ethiopia, which is crucial for reducing childhood morbidity and mortality.

## METHODS

### Study design and setting

We conducted a prospective, observational cohort study on children aged 2–59 months with chest indrawing pneumonia who presented at primary healthcare facilities in five rural districts of the North Gondar zone, Amhara region, Ethiopia, from November 2022 to November 2023. The zone has 24 *woredas* (*i.e.* districts) and an estimated population of 4 066 517 [15]. The study was conducted at five health centres (HCs) (Dabat, Wokin, Dara, Gedebge, and Amba Giorgis), each with a catchment population of about 25 000 (about 5000 households). The HCs provide 24-hour outpatient services and no inpatient care. Two clinical nurses worked in the under-five clinics within the HCs. They were equipped with respiratory rate timers, thermometers, weighing scales, and height-measuring scales (*i.e.* stadiometers). All HCs, meanwhile, had no pulse oximeters and oxygen therapy available. We published more details about the methods used in this study elsewhere [16].

### Study population

After obtaining informed written consent from parents or guardians, we enrolled children aged 2–59 months living in the study catchment area who presented at the selected HCs with cough or breathing difficulty and had chest indrawing. We excluded children if they had severe pneumonia with any general danger signs (convulsions, inability to drink or breastfeed, persistent vomiting, lethargy, unconsciousness) [11], if there was stridor in a calm child if they lived in an area where follow-up was not feasible, or if they were currently enrolled in another study.

### IMCI refresher training

Before enrolment began, all clinical nurses working at the under-five clinics at selected HCs and the independent outcome data collectors underwent a two-day refresher training on using the IMCI chart booklet which was delivered by the study team's paediatrician. The training comprised lectures and video demonstrations in a classroom setting and practical sessions in a hospital. It included classifying children aged 2–59 months presenting with cough or difficult breathing, and practical identification of chest indrawing pneumonia. Additionally, the independent outcome data collectors were oriented to the study's objectives, design, methods, consent forms, and ethical considerations regarding data collection.

### Participant selection and recruitment

The recruitment process involved an initial assessment, enrolment, and subsequent follow-up on day 15 after enrolment.

### Initial assessment and enrolment

During initial assessment, healthcare providers assessed all children aged 2–59 months presenting with cough or difficulty breathing using the Ethiopian IMCI chart booklet [11]. They categorised children into having either cough or cold/no pneumonia, fast breathing pneumonia (≥50 breaths per minute in 2–11-month-old infants and ≥40 breaths per minute in 12–59-month-old children), chest indrawing pneumonia, or severe pneumonia. For eligible children (those with cough, difficulty breathing, and chest indrawing pneumonia), consent for participation and follow-up was obtained from the parent or guardian. Healthcare providers then recorded mandatory patient information on a study form, which included the child's name, age, address, the caregiver's phone number, clinical findings, diagnosis, and treatment. They used the IMCI approach to manage children with chest indrawing pneumonia. The healthcare provider counselled parents and guardians on treatment adherence and when to return to the HC.

### Follow-up and outcome assessment

On day 15 after enrolment, an independent outcome data collector hired and trained specifically for this study recorded the outcomes after interviewing the caregivers. This person was not involved in diagnosing and treating the enrolled children and contacted the parents or caregivers to complete the follow-up. The outcome assessment follow-up visit was completed either at the health facility or the child's home. During the visit, the independent outcome data collector gathered information regarding the child's survival status and whether anyone had received additional outpatient or inpatient treatment during the current illness. They also collected treatment information, including the name of the medication, administration route, duration, and frequency per day. The caregivers provided this information, or it was obtained from medical records if the child had visited the hospital. The window period for the follow-up visit was within a two-day range. In case of a child's death, they performed a verbal autopsy to determine the apparent cause of death.

### Study outcomes

The primary outcome was the survival status of children aged 2–59 months who presented at a primary healthcare facility with chest indrawing pneumonia. Secondary outcomes were the prevalence of antibiotic use and the proportion of hospital admissions. For analysis purposes, we defined a poor clinical outcome as a child who died or was hospitalised at any point between day two (the day after enrolment) and day 15 or who was hospitalised at the time of follow-up. We defined a good clinical outcome as the patient being alive on day 15 and not being taken to the hospital between days two and 15. We recorded the outcome information as described by the caregiver.

### Sample size determination

We calculated the sample size for the descriptive analysis to be 292, based on assumptions of a 5% case fatality risk (CFR) for chest indrawing. Due to a lack of local data for Ethiopia, we used data from a primary care study conducted in Malawi to calculate our sample size. This decision was justified by the contextual similarities between the two countries in terms of healthcare settings and population characteristics. Therefore, assuming a 95% confidence level and a 3% margin of error, and accounting for a 5% loss to follow-up, we calculated the required sample size to be 310 children aged 2–59 months with chest indrawing pneumonia were required for enrolment and follow-up [16].

### Data analysis

We described categorical variables (sex, clinical signs, treatment received, and vital status) with frequencies and percentages. Additionally, we described continuous variables, such as age and duration of treatment, using means and standard deviations (SDs) or medians and interquartile ranges, depending on the distribution of the data. We calculated the CFR, expressed as a percentage, by dividing the number of deaths by day 15 after enrolment by the total number of children with chest indrawing pneumonia enrolled and followed up. We calculated a 95% confidence interval (CI) for the CFR using the standard error. We selected independent variables with established associations from previous studies for analysis to determine the factors associated with poor clinical outcomes. However, due to the low number of cases with poor treatment outcomes, we were unable to conduct a comparative analysis to identify these factors. We cleaned the data to identify missing values, eliminate duplicates, and resolve inconsistencies. 

We performed these analyses using the Stata, version 17 (StataCorp LLC, Texas, USA).

## RESULTS

### Enrolment and baseline characteristics

**Figure 1:**
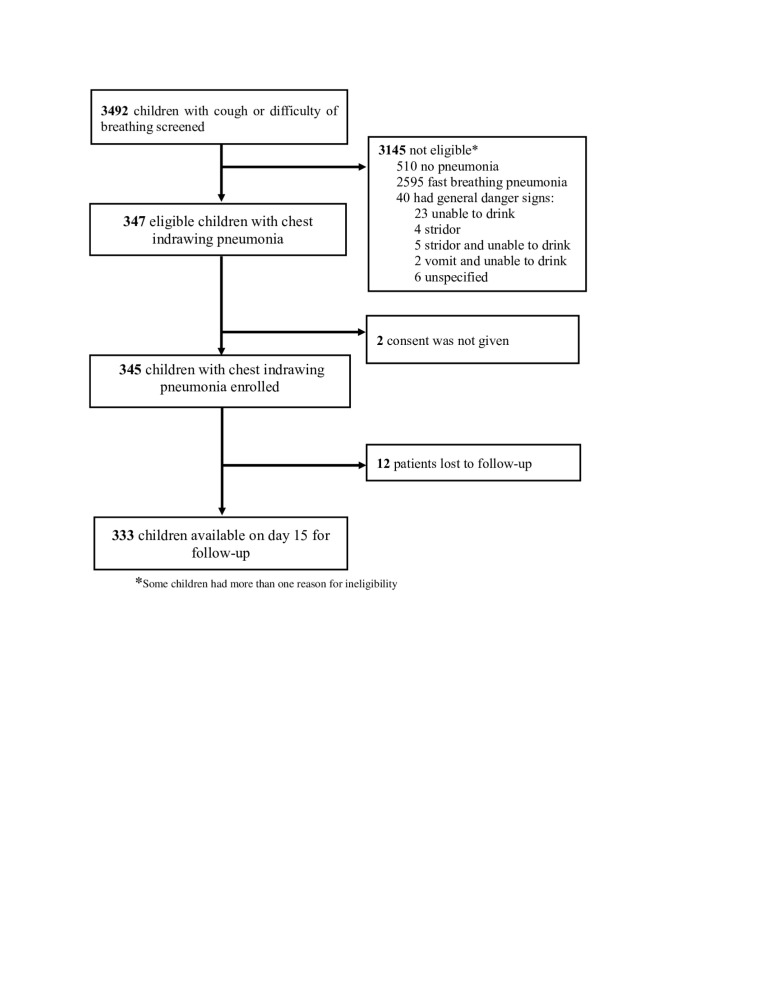
Screening, enrolment, and follow-up of children aged 2–59 months with chest indrawing pneumonia.

We screened 3492 children aged 2–59 months who presented with cough or difficult breathing. Of these, 347 (11.0%) had chest indrawing pneumonia and were further evaluated for enrolment, with 345 (99.4%) remaining for inclusion due to satisfying our eligibility criteria and their parents consenting to participation. We followed up 333 (96.5%) children on day 15 to collect information about the study outcome data among all enrolled children, meaning that only 12 (3.5%) children were lost to follow-up (**Figure 1**).

The children's mean age was 16.9 months (SD = 12.8), indicating a broad age range with a larger number of younger children. Over a half (n = 207; 60.0%) were male and one-third were enrolled from the Amba Giorgis district. Eighteen children had a weight-for-height (WHZ) ≤−3 SD, 27 had a weight-for-age (WAZ) ≤−3 SD, and 81 had a height-for-age (HAZ) ≤−3 SD, indicating a substantial burden of chronic malnutrition, but not severe acute malnutrition. Over half of the children (n = 176; 51.0%) were either partially or not vaccinated, suggesting a significant immunisation gap in immunisation coverage within the study population (**Table 1**).

**Table 1:** Characteristics of children aged 2–59 months with chest indrawing pneumonia

	n (%)
**Total number of participants**	345
**Enrolment site districts**	
Ambagiorgis	112 (32.5)
Dabat	76 (22.0)
Dara	8 (2.3)
Gedebge	59 (17.1)
Woken	90 (26.1)
**Maternal education**	
None	178 (51.6)
Able to read and write	33 (9.6)
Primary	96 (27.8)
Secondary and above	26 (7.5)
Unknown†	12 (3.5)
**Age of children, in months (x̄ = 16.9, SD = 12.8; MD = 13, IQR = 7–23)**	
2–11	147 (42.6)
12–23	112 (32.5)
24–59	86 (24.9)
**Sex**	
Male	207 (60.0)
Female	138 (40.0)
**Respiratory rate, breaths/min**	
2–11 mo (x̄ = 58.2, SD = 5.7; MD = 57, IQR = 54–60)	
*50–59*	104 (30.1)
*≥60*	43 (12.5)
12–59 mo (x̄ = 53.4, SD = 7.6; MD = 50.5, IQR = 48–58)	
*40–49*	86 (24.9)
*≥50*	112 (32.5)
**MUAC, in cm**	
<11.5	9 (2.7)
11.5–12.5	45 (13.0)
>12.5	225 (65.2)
NA*	66 (19.1)
**HAZ, Z-score**	
≥−2	191 (55.4)
−2 to −3	73 (21.1)
≤−3	81 (23.5)
**WAZ, Z-score**	
≥−2	266 (77.1)
−2 to −3	52 (15.1)
≤−3	27 (7.8)
**WHZ, Z-score**	
≥−2	299 (86.7)
−2 to −3	28 (8.1)
≤−3	18 (5.2)
**Immunisation status**	
Unvaccinated	12 (3.5)
Partially vaccinated	164 (47.5)
Fully vaccinated or vaccinated for age	157 (45.5)
Unknown†	12 (3.5)

HAZ – height for age, IQR – interquartile range, MD – median, MUAC – mid-upper arm circumference, SD – standard deviation, WAZ – weight for age, WHZ – weight for height, x̄ – mean

*Children below six months of age.

†Lost to follow-up.

### Outcomes

Two deaths occurred among the 333 children followed up on day 15, resulting in a CFR of 0.6% (95% CI = 0.35, 0.85) (**Box 1**).

Box 1 **Verbal autopsy details for deaths (n = 2)**

Case 1: A 54-month-old male child had chest indrawing pneumonia with a respiratory rate of 58 breaths per minute, with no general danger signs. He was partially vaccinated, had both severe wasting (WHZ = −3.7) and severe stunting (HAZ = −3.3), and was severely underweight (WAZ = −4.1) at enrolment. After one day of illness, he developed upper abdominal cramps, and the fast breathing progressed to difficulty in breathing. The next day, *i.e.* after two days of illness, he developed bloody vomiting and fever, though he did not have diarrhoea. According to the caregiver's report, the child also had sunken eyes and lethargy. His condition rapidly deteriorated on the day of his death, leading to unconsciousness seven hours before he passed away at home on the second day of enrolment and the fourth day of illness. Unfortunately, he did not receive any further treatment from the home, community, health centre, or hospital. The family did not seek medical care; they were waiting instead for the appointment given previously by the healthcare professional. It remains unrecorded whether he was still on oral amoxicillin at the time of his death.

Case 2: A 5-month-old female infant presented with lower chest indrawing, a respiratory rate of 55 breaths per minute, and no danger signs at enrolment. She was severely stunted (HAZ = −6.6) and wasted (WHZ = 6.6) and was partially vaccinated. The day following the enrolment, the infant's feeding was reported as decreased, and she reportedly developed an ‘abdominal cramp’ 1–2 hours before her death. According to the grandmother's report, the infant deteriorated rapidly and passed away on the second day of enrolment. The family had not sought medical care from the community, health centre, or hospital. They believed that the cause of death was the ‘evil eye’ and the rapid progression of her condition. It is not recorded whether she was still taking amoxicillin at the time of her death.

According to mothers or caregivers, 322 out of 331 (97.3%) survivors were reported as cured, eight (2.4%) were the same as at enrolment, and one child’s condition had worsened. Four out of the eight patients reported having the ‘same’ condition, along with one patient whose condition was ‘worse’ and who had been taken to the hospital for further care.

Among 333 children followed up on day 15, 13 (3.9%) had visited a hospital between the second day of enrolment and day 15. Six patients (1.8%) were treated as outpatients, while the remaining seven (2.1%) were admitted. Five of the six children treated as outpatients received oral medications (amoxicillin and azithromycin), while one patient received injectable ceftriaxone. All admitted patients received injectable medications, either alone or in combination (**Table 2**).

**Table 2:** Antibiotic medications administered to enrolled children who visited a hospital (n = 13)

	Duration, in days	Number of patients
**Inpatient treatment**		
Injectable ceftriaxone alone	2–4	5
Injectable ampicillin plus gentamicin	5	1
Injectable ampicillin plus gentamicin, followed by injectable ceftriaxone	5, 5†	1
**Outpatient treatment**		
Injectable ceftriaxone with oral amoxicillin	1, 5†	1
Oral amoxicillin*	5	2
Oral azithromycin	3	3

*These patients discontinued the oral amoxicillin prescribed at the health centre before a hospital visit.

†Duration of administration for the second antibiotic.

### Treatment of chest indrawing pneumonia

All 345 enrolled children were prescribed oral amoxicillin on an outpatient basis. The majority (n = 340, 98.6%) were prescribed a five-day course of oral amoxicillin, while the remaining five patients received a seven-day course. No injectable medications were administered at the time of enrolment. Oral amoxicillin to be administered in two divided doses was provided either in dispersible tablet form (125 mg or 250 mg) to 120 patients (34.8%) or as a suspension (125 mg/5 ml or 250 mg/5 ml) for 221 patients (64.1%). The formulation type was not documented for four patients.

### Adherence to treatment

Full treatment adherence to the prescribed oral amoxicillin was reported for 315 of 333 (94.6%) children who were followed up on day 15 (**Table 3**). The reasons for not completing the prescribed course of oral amoxicillin in 18 (5.4%) children were: lack of improvement in their condition (n = 12), improvement before completing the course of antibiotics (n = 4), and death (n = 2). Non-adherent patients (n = 12) who did not improve were taken to the hospital between the day of enrolment and day 15, where they were prescribed other antibiotics.

**Table 3:** Adherence to oral amoxicillin in enrolled children

	n (%)
**Adherence to oral antibiotics (n = 333)**	
Yes	315 (94.6)
No	18 (5.4)
**Reason for non-adherence (n = 18)**	
Not improved and taken to hospital	12 (66.7)
Improved and stopped oral antibiotic	4 (22.2)
Death	2 (11.1)

### Factors associated with the treatment outcome of chest indrawing pneumonia

Eight of 15 children (53%) with poor clinical outcomes were aged 2–11 months (Table S1 in the **Online Supplementary Document**). Only three (20%) children with poor treatment outcomes had received full vaccination according to their age.

## DISCUSSION

Our study showed that managing chest indrawing pneumonia on an outpatient basis by IMCI-trained health workers in primary healthcare facilities in Ethiopia resulted in only two deaths and seven hospitalisations. All enrolled children were prescribed oral amoxicillin, and an overwhelming majority (94.6%) adhered to the prescribed regimen. These results provide empirical evidence in support of the current treatment approach for managing chest indrawing pneumonia in ambulatory settings.

The low mortality observed here is consistent with results from other studies conducted in similar settings where oral amoxicillin was used for the home treatment of chest indrawing pneumonia. For instance, it aligns with the findings of two observational studies of outpatient treatment for chest indrawing pneumonia with oral amoxicillin, where no deaths were reported: one study was multicentre, conducted in Bangladesh, Egypt, Ghana, and Vietnam [17], while the other was conducted in Papua New Guinea [18]. Another observational ambulatory treatment study in Kenya also reported a CFR of 0.2% [19]. Similarly, three community-based randomised clinical trials of ambulatory treatment reported comparable results: 0.2% CFR in the multi-country, multicentre enhanced community case management to increase access to pneumonia treatment study [20], 0.05% CFR in Haripur, Pakistan, [21] and 0.5% CFR in Matiari, Pakistan [22]. In addition, certain hospital-based randomised clinical trials also reported a CFR lower than 1%. These include the multi-country, multicentre amoxicillin penicillin pneumonia international study [23], the new outpatient short-course home oral therapy for severe pneumonia study in Pakistan [24], and a randomised clinical trial conducted in Kenya in hospitalised patients [25]. However, higher CFRs were observed in a multicentre hospital-based study from Kenya (5.9%) [14] and Ethiopia (5.3%) [26], which might be attributed to differences in the severity of the disease, as the studies included hospitalised children with both chest indrawing pneumonia and pneumonia with danger signs. The low case fatality observed in our study might be related to timely care-seeking behaviour, effective implementation of simplified antibiotic regimens, or strong health worker engagement. Conversely, delays in recognising danger signs, challenges accessing referral care, or underlying childhood vulnerabilities could contribute to poor outcomes in other contexts. Although we cannot mechanistically explain the low CFR due to the observational nature of our study, our findings can guide future hypothesis-driven research to explore biological, behavioural, healthcare, and programme-related factors.

Our study demonstrated high adherence among health workers to the IMCI protocol for managing chest indrawing pneumonia and good adherence to the treatment recommendations by parents. Our high adherence rate is comparable to that of a previous study [17], which also reported a 95% adherence rate, higher than the 84% adherence rate observed at the community level in the community-based pneumonia management study [20]. The high adherence rate here may be attributed to the supportive supervision and adherence counselling sessions provided during the study period.

The outpatient treatment of chest indrawing pneumonia offers substantial benefits for the health system, the family, and the child, especially in resource-scarce settings. Our data thus has several implications for national health policy and programmes. From a health system perspective, the outpatient management of pneumonia could potentially result in a 40% reduction in the treatment cost, saving up to USD 1.16 billion in the 74 countdown countries [12]. At the institutional level, outpatient management of pneumonia alleviates pressure on hospital resources [27], including bed space, medical staff, and equipment. For the family, outpatient treatment minimises disruptions to daily life, reduces the financial burden associated with admission and length of hospital stays, such as costs for meals, transportation, and potential loss of income [13,28–30]. These benefits are particularly crucial in resource-limited settings where healthcare resources are scarce, and the economic burden on families could be substantial [31].

In addition to the economic benefits, outpatient care for chest indrawing pneumonia has substantial implications for the health system by reducing hospitalisations and the risk of healthcare-associated infections (HAIs), which otherwise result in substantial morbidity and mortality in low- and middle-income countries [32–34]. An analysis of data from 99 countries reported 136 million episodes of HAIs globally per year [35]. Antibiotic-resistant organisms are more likely to cause HAIs, resulting in severe complications and protracted hospitalisation needing sophisticated and risky medical interventions that increase healthcare costs [36,37]. The development of antimicrobial resistance (AMR) is an enormously growing healthcare challenge, especially in low- and middle-income countries [38,39], and predominantly occurs in hospital settings rather than community settings [40,41]. The overuse and misuse of antibiotics have led to a growing threat of AMR [42], which limits the effectiveness of essential antibiotics and threatens the success of medical interventions [43]. Addressing AMR is critical to improving healthcare in low- and middle-income countries and requires rational antibiotic use and investments in infection control and surveillance. By minimising hospital admissions, outpatient treatment can decrease exposure to these risks. Additionally, the WHO standard case management of pneumonia promotes the rational use of antibiotics [44], while IMCI case management training and implementation improve the rational use of antimicrobial drugs [45]. Thus, outpatient management aligns with broader healthcare goals of reducing AMR and improving the overall quality of healthcare in resource-limited settings.

Our study has several strengths. We implemented it in an established programme setting within the health system, reflecting a real-world scenario. Notably, there was no withdrawal of consent; we lost only a few patients to follow-up, and adherence to IMCI recommendations was high. However, it also had some limitations. One of these was the recall bias, as we gathered the information from mothers or caregivers after a two-week period had passed. Additionally, the outcomes were based on caregiver reports, which might have introduced some bias. In general, caregiver bias is more pronounced in subjective domains than in observable findings, and one would expect a parent or mother to report that the child is still sick rather than getting better. Finally, due to security concerns and financial constraints, we had to limit the inclusion of enrolled children to areas where follow-up was feasible; this might have systematically excluded geographically marginalised populations. We cannot exclude the impact of unmeasured confounding variables due to the study's observational nature.

## CONCLUSIONS

We found that chest indrawing pneumonia without danger signs in children aged 2–59 months can be effectively managed with a five-day course of oral amoxicillin in ambulatory settings, resulting in very low mortality, low hospitalisation rates, and high treatment adherence. Overall, outpatient treatment enhances the efficiency and sustainability of healthcare services, supports a more equitable distribution of resources, and promotes a patient-centric approach to managing pneumonia, thereby improving health outcomes and strengthening the resilience of the healthcare system. Based on this result, we recommend the widespread adoption and implementation of these guidelines, particularly in settings where access to a health facility is limited and a referral is not feasible. The practitioners can confidently manage chest indrawing pneumonia in children on an outpatient basis with oral antibiotics. Multicentre implementation studies can further explore the enablers and challenges of effectively implementing these recommendations, providing valuable insights into real-world applicability and sustainability.

## Additional material


Online Supplementary Document
