# Supplementary material for: Management and outcomes of chest-indrawing pneumonia among children aged 2–59 months in a programme setting in Ethiopia: a prospective observational study
Source: J Glob Health. 2025 Aug 4;15:04217. doi: 10.7189/jogh.15.04217 (PMC12319351; doi:10.7189/jogh.15.04217)
Supplement: Online Supplementary Document [file jogh-15-04217-s001.pdf]

Supplement to: Tigabu Z, Toni AT, Guadu T, Yilma TM, Awoke T, Engdaw GT, Tazebew A, Qazi S, Nisar YB. Management and outcomes of chest-indrawing pneumonia among children aged 2–59 months in a programme setting in Ethiopia: a prospective observational study. J Glob Health. 2025;15:04217.

Table S1: Characteristics of children with chest indrawing pneumonia according to their treatment outcomes (N=333)

Variables	Poor outcome* (n=15) n (%)	Good outcome (n=318) n, (%)
**District**		
Ambagiorgis	5 (33.3)	101 (31.8)
Dabat	4 (26.7)	72 (22.6)
Dara	0 (0.0)	8 (2.5)
Gedebge	5 (33.3)	50 (15.7)
Woken	1 (6.7)	87 (27.4)
**Age (months)**		
2-11	8 (53.3)	134 (42.1)
12-23	3 (20.0)	106 (33.3)
24-59	4 (26.7)	78 (24.5)
**Sex**		
Male	10 (66.7)	190 (59.7)
Female	5 (33.3)	128 (40.3)
**Respiratory rate**		
2-11 months, >= 50	8 (53.3)	134 (42.1)
12-59 months, >= 40	7 (46.7)	184 (57.9)
**MUAC in cm**		
<11.5	0 (0.0)	9 (2.8)
11.5-12.5	6 (40.0)	29 (9.1)
>=12.5	6 (40.0)	221 (69.5)
NA[†]	3 (20.0)	59 (18.6)
**Height for age (Z score)**		
≥ - 2	8 (53.3)	176 (55.3)
- 2 to – 3	3 (20.0)	67 (21.1)
≤ -3	4 (26.7)	75 (23.6)
**Weight for age (Z score)**		
≥ - 2	10 (67.7)	247 (77.7)
- 2 to – 3	2 (13.3)	47 (14.8)
≤ -3	3 (20.0)	24 (7.5)
**Weight for height (Z score)**		
≥ - 2	13 (86.7)	276 (86.8)
- 2 to – 3	1 (6.7)	27 (8.5)
≤ -3	1 (6.7)	15 (4.7)
**Maternal education**		
None	7 (46.7)	115 (36.2)
Educated	8 (53.3)	203 (63.8)
**Immunisation status**		
Unvaccinated	1 (6.7)	11 (3.5)
Partially vaccinated.	11 (73.3)	153 (48.1)
Fully vaccinated for age	3 (20.0)	154 (48.4)

*15 poor clinical outcomes included two deaths and 13 patients who were taken to the hospital, good clinical outcome was defined as children who were alive and had not been hospitalised.

† Infants below 6 months. MUAC: Mid-Upper Arm Circumference.
